# Three-Country Snapshot of Ornithine Transcarbamylase Deficiency

**DOI:** 10.3390/life12111721

**Published:** 2022-10-27

**Authors:** Berna Seker Yilmaz, Julien Baruteau, Nur Arslan, Halil Ibrahim Aydin, Magalie Barth, Ayse Ergul Bozaci, Anais Brassier, Ebru Canda, Aline Cano, Efstathia Chronopoulou, Grainne M. Connolly, Lena Damaj, Charlotte Dawson, Dries Dobbelaere, Claire Douillard, Fatma Tuba Eminoglu, Sahin Erdol, Melike Ersoy, Sherry Fang, François Feillet, Gulden Gokcay, Emine Goksoy, Magali Gorce, Asli Inci, Banu Kadioglu, Fatih Kardas, Cigdem Seher Kasapkara, Gonca Kilic Yildirim, Deniz Kor, Melis Kose, Cecilia Marelli, Helen Mundy, Siobhan O’Sullivan, Burcu Ozturk Hismi, Radha Ramachandran, Agathe Roubertie, Mehtap Sanlilar, Manuel Schiff, Srividya Sreekantam, Karolina M. Stepien, Ozlem Uzun Unal, Yilmaz Yildiz, Tanyel Zubarioglu, Paul Gissen

**Affiliations:** 1 Genetics and Genomic Medicine Department, Great Ormond Street Institute of Child Health, University College London, London WC1N 1EH, UK; 2 National Institute of Health Research Great Ormond Street Biomedical Research Centre, London WC1N 1EH, UK; 3 Metabolic Medicine Department, Great Ormond Street Hospital for Children NHS Foundation Trust, London WC1N 3JH, UK; 4 Paediatric Metabolic Medicine Department, Dokuz Eylul University Faculty of Medicine, Izmir 35340, Turkey; 5 Paediatric Metabolic Medicine Department, Baskent University Faculty of Medicine, Ankara 06490, Turkey; 6 Centre de Référence des Maladies Héréditaires du Métabolisme, CHU Angers, 4 rue Larrey, CEDEX 9, 49933 Angers, France; 7 Paediatric Metabolic Medicine Department, Diyarbakir Children’s Hospital, Diyarbakir 21100, Turkey; 8 Reference Center for Inborn Errors of Metabolism, Necker University Hospital, APHP and University of Paris Cité, 75015 Paris, France; 9 Paediatric Metabolic Medicine Department, Ege University Faculty of Medicine, Izmir 35100, Turkey; 10 Reference Center of Inherited Metabolic Disorders, Timone Enfants Hospital, 264 rue Saint-Pierre, 13005 Marseille, France; 11 Department of Inherited Metabolic Disease, Division of Women’s and Children’s Services, University Hospitals Bristol NHS Foundation Trust, Bristol BS1 3NU, UK; 12 Belfast Health and Social Care Trust, Belfast BT9 7AB, UK; 13 Centre de Compétence Maladies Héréditaires du Métabolisme, CHU Hôpital Sud, CEDEX 2, 35203 Rennes, France; 14 Metabolic Medicine Department, University Hospitals Birmingham NHS Foundation Trust, Birmingham B15 2GW, UK; 15 Medical Reference Center for Inherited Metabolic Diseases, Jeanne de Flandre University Hospital and RADEME Research Team for Rare Metabolic and Developmental Diseases, EA 7364 CHRU Lille, 59000 Lille, France; 16 Paediatric Metabolic Medicine Department, Ankara University Faculty of Medicine, Ankara 06080, Turkey; 17 Paediatric Metabolic Medicine Department, Uludag University Faculty of Medicine, Bursa 16059, Turkey; 18 Paediatric Metabolic Medicine Department, Dr Sadi Konuk Reseach & Training Hospital, Istanbul 34450, Turkey; 19 Centre de Référence des Maladies Métaboliques de Nancy, CHU Brabois Enfants, 5 Rue du Morvan, 54500 Vandœuvre-lès-Nancy, France; 20 Paediatric Metabolic Medicine Department, Istanbul University Istanbul Faculty of Medicine, Istanbul 34093, Turkey; 21 Paediatric Metabolic Medicine Department, Cengiz Gokcek Children’s Hospital, Gaziantep 27010, Turkey; 22 Centre de Référence des Maladies Rares du Métabolisme, Hôpital des Enfants—CHU Toulouse, 330 Avenue de Grande-Bretagne, CEDEX 9, 31059 Toulouse, France; 23 Paediatric Metabolic Medicine Department, Gazi University Faculty of Medicine, Ankara 06500, Turkey; 24 Paediatric Metabolic Medicine Department, Konya City Hospital, Konya 42020, Turkey; 25 Paediatric Metabolic Medicine Department, Erciyes University Faculty of Medicine, Kayseri 38030, Turkey; 26 Paediatric Metabolic Medicine Department, Ankara Yildirim Beyazit University Faculty of Medicine, Ankara 06800, Turkey; 27 Paediatric Metabolic Medicine Department, Osmangazi University Faculty of Medicine, Eskisehir 26480, Turkey; 28 Paediatric Metabolic Medicine Department, Cukurova University Faculty of Medicine, Adana 01250, Turkey; 29 Paediatric Metabolic Medicine Department, Faculty of Medicine, Izmir Katip Celebi University, Izmir 35620, Turkey; 30 MMDN, University Montpellier, EPHE, INSERM, 34090 Montpellier, France; 31 Expert Center for Metabolic and Neurogenetic Diseases, Centre Hospitalier Universitaire (CHU), 34090 Montpellier, France; 32 Evelina Children’s Hospital, Guy’s and St Thomas’ NHS Foundation Trust, London SE1 7EH, UK; 33 Royal Belfast Hospital for Sick Children, Belfast BT12 6BA, UK; 34 Paediatric Metabolic Medicine Department, Marmara University Faculty of Medicine, Istanbul 34854, Turkey; 35 Guy’s and St Thomas’ NHS Foundation Trust, London SE1 7EH, UK; 36 Paediatric Metabolic Medicine Department, Antalya Training and Research Hospital, Antalya 07100, Turkey; 37 Birmingham Women’s and Children’s Hospital NHS Foundation Trust, Birmingham B4 6NH, UK; 38 Adult Inherited Metabolic Diseases, Salford Royal NHS Foundation Trust, Salford M6 8HD, UK; 39 Paediatric Metabolic Medicine Department, Kocaeli University Faculty of Medicine, Kocaeli 41380, Turkey; 40 Paediatric Metabolic Medicine Department, Hacettepe University Faculty of Medicine, Ankara 06230, Turkey; 41 Paediatric Metabolic Medicine Department, Istanbul University-Cerrahpasa Faculty of Medicine, Istanbul 34096, Turkey

**Keywords:** ornithine transcarbamylase deficiency, hyperammonaemia, neonatal-onset, late-onset, asymptomatic, protein restriction, ammonia scavengers, liver transplantation

## Abstract

X-linked ornithine transcarbamylase deficiency (OTCD) is the most common urea cycle defect. The disease severity ranges from asymptomatic carrier state to severe neonatal presentation with hyperammonaemic encephalopathy. We audited the diagnosis and management of OTCD, using an online 12-question-survey that was sent to 75 metabolic centres in Turkey, France and the UK. Thirty-nine centres responded and 495 patients were reported in total. A total of 208 French patients were reported, including 71 (34%) males, 86 (41%) symptomatic and 51 (25%) asymptomatic females. Eighty-five Turkish patients included 32 (38%) males, 39 (46%) symptomatic and 14 (16%) asymptomatic females. Out of the 202 UK patients, 66 (33%) were male, 83 (41%) asymptomatic and 53 (26%) symptomatic females. A total of 19%, 12% and 7% of the patients presented with a neonatal-onset phenotype in France, Turkey and the UK, respectively. Vomiting, altered mental status and encephalopathy were the most common initial symptoms in all three countries. While 69% in France and 79% in Turkey were receiving protein restriction, 42% were on a protein-restricted diet in the UK. A total of 76%, 47% and 33% of patients were treated with ammonia scavengers in Turkey, France and the UK, respectively. The findings of our audit emphasize the differences and similarities in manifestations and management practices in three countries.

## 1. Introduction

Ornithine transcarbamylase (OTC) deficiency (OTCD) [MIM: 311250] is an X-linked defect of ureagenesis and the most common urea cycle disorder (UCD), accounting for about half of the reported patients [1]. The prevalence of OTCD was estimated to be between 1 in 14,000 to 1 in 80,000 in earlier publications [2,3]. More recent studies based on newborn screening programmes and disease registries identified an incidence of 1 in 62,000 in Finland, 1 in 63,000 in USA and 1 in 69,904 in Italy [4,5,6].

OTC (EC 2.1.3.3), encoded by the OTC gene located on the short arm of the X chromosome (Xp11.4), is a homotrimeric, mitochondrial enzyme expressed in the liver and intestine. This catalyzes the transfer of a carbamoyl group from carbamoyl phosphate to the amino group of L-ornithine, yielding citrulline and phosphate as a part of the main pathway in ammonia clearance [7]. Therefore, profound hyperammonaemia, decreased citrulline, elevated glutamine and urinary orotic acid levels are the biochemical hallmarks of the disorder. Clinical signs and symptoms are caused by the toxic effects of hyperammonaemia and high brain glutamine levels on the central nervous system, leading to mental status changes, seizures, cerebral oedema and, in severe cases, death [8]. Acute hyperammonaemia may also cause liver dysfunction [9].

OTCD has a wide phenotypic heterogeneity, due to the type of mutation, genetic background and environment, heterozygous females may be asymptomatic depending on the level of X-inactivation [10,11]. OTC-deficiency patients can present with either a neonatal onset phenotype usually seen in hemizygous males that completely abrogates enzyme activity resulting from null alleles or a later onset phenotype with residual enzyme activity that can be seen in heterozygous females and some male patients [12]. Heterozygous females are symptomatic in approximately 20% of cases with a highly variable age of onset and clinical features [13]. Chronic manifestations include protein aversion, recurrent vomiting, developmental delay, psychiatric disorders and variable degrees of liver dysfunction [14,15].

The diagnosis of OTC deficiency in neonatal onset cases may be straightforward, with biochemical findings including elevated glutamine, very low/absent citrulline and elevated urinary orotic acid [16]. However, diagnosis may be more challenging in late onset presentations and asymptomatic ones. Mutation identification is the gold-standard method to confirm diagnosis, allowing for prenatal testing, carrier identification and even genotype–phenotype correlation [14,16]. More than 400 disease-causing mutations have been reported in the *OTC* gene. [10] However, nearly 20% of individuals in whom a diagnosis of OTCD is made based on reduced enzyme activity do not have an identifiable pathogenic variant in the exons or exon–intron boundaries of *OTC* gene [10]. In these individuals, variants may be located in the regulatory regions, such as promoters or enhancers, or deep intronic regions (i.e., beyond approximately 20–30 base pairs from the exon–intron boundary). Liver-tissue-derived RNA-based studies or the use of oligonucleotide array comparative genomic hybridization (CGH) can successfully increase diagnostic success in these patients [17,18]. Although liver or intestinal mucosa OTC enzymatic activity assays can also be helpful for diagnostic confirmation, this may be normal in an affected female, depending upon the pattern of X-inactivation in the liver.

The standard of care consists of a protein-restricted diet, daily ammonia scavengers, and arginine/citrulline supplementation. Adequate protein and energy supply can be based on The Food and Agriculture Organization of the United Nations (FAO)/The World Health Organization (WHO)/United Nations University (UNU) 2007 “safe levels of protein intake”, which allows for optimal growth and metabolic stability. Sodium benzoate, sodium phenylbutyrate and glycerol phenylbutyrate are the available ammonia scavengers for management at present [16]. However, this treatment does not prevent acute hyperammonaemia episodes provoked by catabolic stress, such as intercurrent illness or fasting, which can cause severe neurological damage, emphasizing the high levels of unmet needs. To date, liver transplantation has been the only curative therapy; however, owing to technical limitations, a shortage of donor livers and the need for lifelong immunosuppression, this is not widely available [19,20]. In the field of cell-based therapies, hepatocyte transplantation has also been suggested as a therapeutic option in recent years. It may be considered when there is an organ donor shortage, and it may be a bridge for a later liver transplantation [21]. OTCD is a paradigm of severe liver-inherited metabolic diseases; many novel therapies have been or are targeting this condition, e.g., adenoviral or adeno-associated viral (AAV) gene therapy, mRNA therapy and gene editing [22,23,24,25,26].

Due to the lack of a sufficient understanding of the natural history of the disease, there is a diversity of approaches to this group of disorders. Therefore, revised guidelines have recently been published for the diagnosis and management of UCDs by a 17-expert panel assembled from eight different European countries and Israel [16]. The goal of these guideline is to enable informed decision-making as an approach for UCD patient care and for it to be considered an evidence-based method to support optimize care. Hence, this audit aimed to measure the current practice within France, Turkey and the UK in terms of the diagnosis and management of OTCD against the recommendations in the most recent guidelines.

## 2. Materials and Methods

We conducted an audit using an online survey containing 12 questions (one question per screen) for data collection. The primary method of accessing and completing the survey was via an online well-known and established online survey platform (SurveyMonkey^®^ (Momentive Inc., San Mateo, CA, USA), www.surveymonkey.com, accessed on 28 June 2021) to facilitate widespread distribution of the survey and ease of data collection. The survey company hosted and collected the survey data and only participants who were sent the email could connect to the link and respond to the questionnaire. The survey company guarantees data encryption and is certified by EU-U.S. Privacy Shield. 

The questionnaire was drafted after a thorough review of the current guideline and evaluated for clarity of questions, appropriateness of responses, and ease of participation. The survey was not validated by a third party. Respondents were asked to consider only alive patients under their medical care. The audit was fully anonymous and, as no individual data were collected, no ethical board approval was required. The complete survey is available in the Supplementary Data (Supplementary Table S1).

The request to participate in the survey was sent by e-mail to the heads of the metabolic units of 75 centres: 40 in Turkey, 24 in France and 11 in the UK. Participation in the audit was voluntary and the online survey was only sent to the participating centres. The head of the centres nominated a delegate person from each centre to respond to the survey in order to prevent duplications. All responses were received in 9 months by June 2022 from the delegate person of each metabolic centre. A three-month period was given for each country. Recurrent reminders were sent until the survey was fully completed by the delegate person. 

All results were downloaded from survey platform website onto an Excel sheet. The obtained data were analysed using the graphical and analytical features of Microsoft Excel. Survey results were presented using descriptive statistics, including count and percentage for categorical variables. 

## 3. Results

In total, there were 39 centres from three countries (9 from France, 21 from Turkey and 9 from the UK). A total of 495 patients followed-up since 2004, including 208 from France, 85 from Turkey and 202 from the UK. Total responses collected in 9 months from September 2021 to June 2022; a three-months period was given for each country. 

### 3.1. Demographic Characteristics of Patients

**Figure 1:**
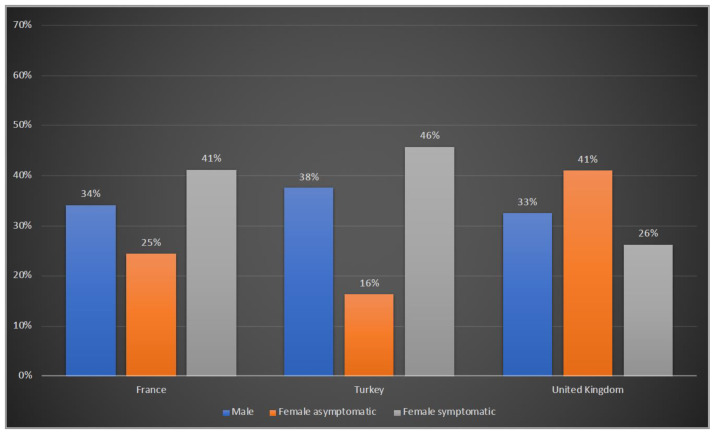
Gender distribution of OTCD patients in France, Turkey and the UK. Blue: male; orange: female asymptomatic; grey: female symptomatic.

Of the 208 patients reported from France, 71 (34%) were males, 51 (25%) were asymptomatic and 86 (41%) were symptomatic females. In Turkey, 32 (38%) patients were males, there were 14 (16%) asymptomatic and 39 (46%) symptomatic female patients. In the UK, 66 (33%) male patients were reported, with the others being 83 (41%) asymptomatic and 53 (26%) symptomatic females (Figure 1). 

**Figure 2:**
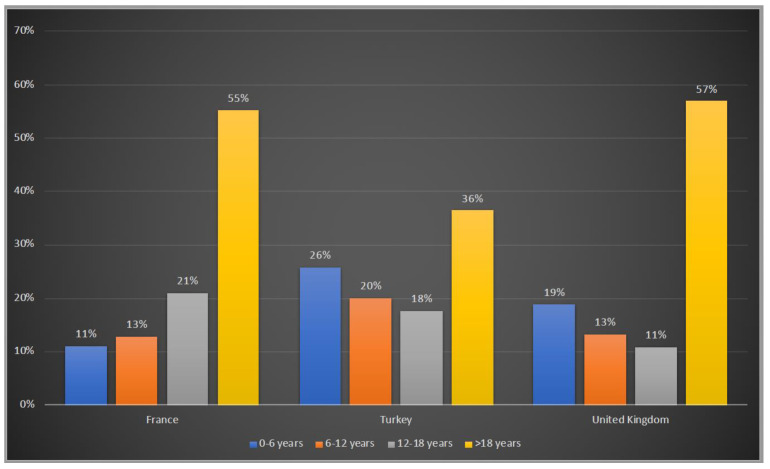
Age groups of OTCD patients in France, Turkey and the UK. Blue:0–6 years old; orange:6–12 years old; grey: 12–18 years old; yellow: >18 years old.

Patients were asked to be included in 4 age groups: 0–6 years, 6–12 years, 12–18 years and >18 years. 45%, 64% and 43% of the patients were under the age of 18 years old in France, Turkey and the UK, respectively whereas Turkey has the highest number of patients in the 0–6 years old group with 26%, followed by the UK with 19% and France with 11% (Figure 2). 

### 3.2. Clinical Presentation

**Figure 3:**
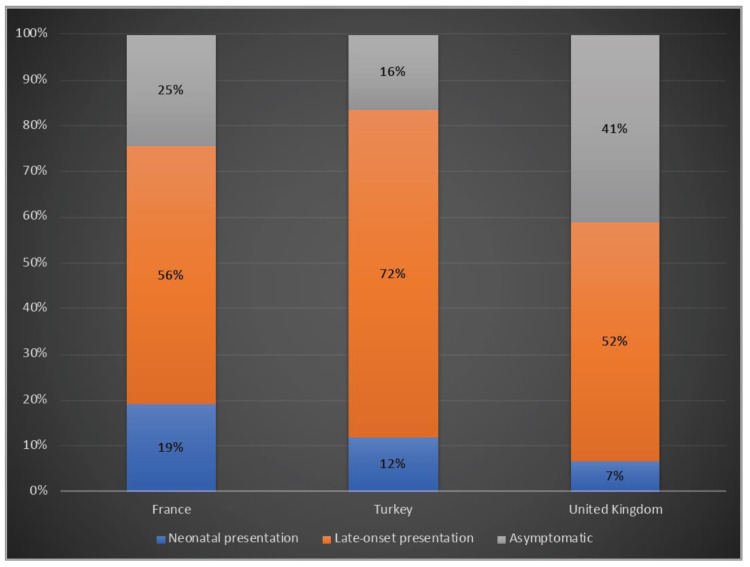
Clinical phenotypes of OTCD patients in France, Turkey and the UK. Blue: Neonatal presentation; orange: late-onset presentation; grey: asymptomatic.

A total of 19%, 12% and 7% of the patients presented with a neonatal onset phenotype, whereas 39%, 42% and 67% had a late-onset presentation and 25%, 16% and 41% were asymptomatic in France, Turkey and the UK, respectively (Figure 3). 

Family history of OTCD was reported in 36% of the patients from France, 45% of the patients from Turkey and 60% of the total number of patients in the UK. 

**Figure 4:**
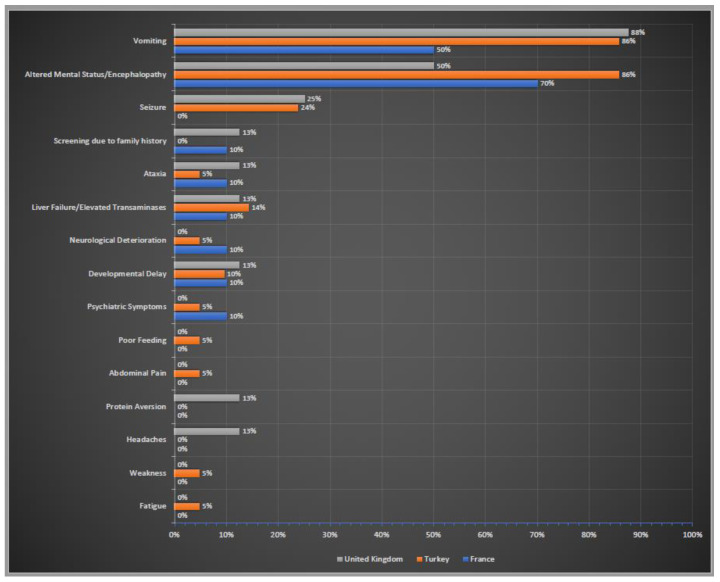
Initial clinical symptoms of OTCD patients in France, Turkey and the UK. Percentages of reported initial clinical symptoms. In blue France; in orange Turkey; in grey the UK.

Initial clinical symptoms of these patients were summarised in Figure 4. Altered neurological status (confusion, lethargy, stupor, coma) and encephalopathy were the most common neurological symptoms in all three countries. The gastrointestinal symptoms also ranked very high in all three cohorts; vomiting was the most prominent symptom. Liver failure/elevated liver transaminases were also reported by the centres in three countries. Screening due to family history was stated only by the centres in France and UK. 

### 3.3. Diagnosis of OTCD Patients

Diagnostic confirmation was established by molecular analysis in almost all patients from three countries (96%, 92% and 97% in France, Turkey and the UK, respectively). Measurement of hepatic or intestinal OTC enzymatic activity was reported in 20% of the patients from France, whereas this only occurred in 6% in Turkey and 7% in the UK. 

### 3.4. Disease Management

**Figure 5:**
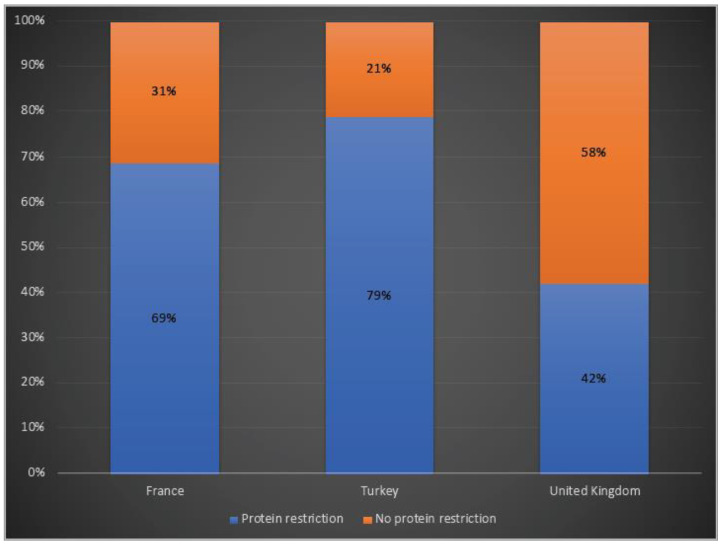
Protein restriction in OTCD patients in France, Turkey and the UK. Blue: Protein restriction; orange: no protein restriction.

While 69% of patients in France and 79% of the ones in Turkey were receiving protein restriction, 42% of the OTCD patients in the UK were on a protein-restricted diet (Figure 5). 

**Figure 6:**
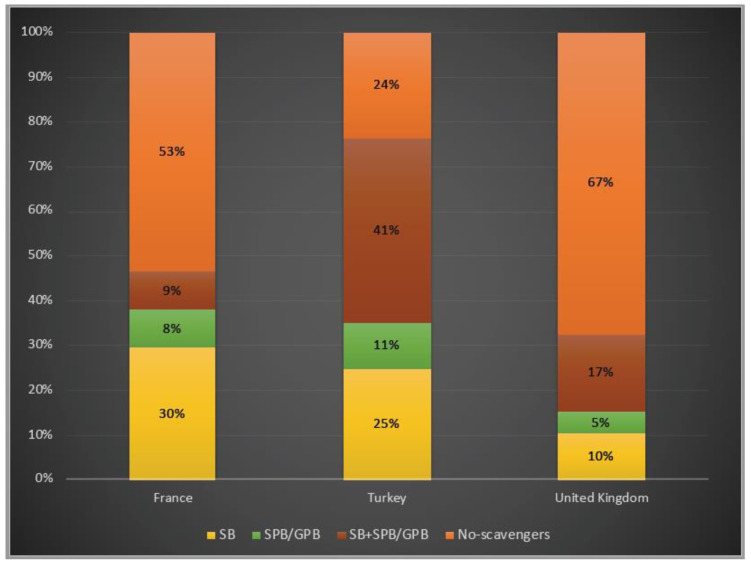
Ammonia scavenger usage in OTCD patients in France, Turkey and the UK. Yellow: sodium benzoate usage; green: sodium/glycerol phenylbutyrate usage; orange–red: dual therapy (sodium benzoate and sodium/glycerol phenylbutyrate) usage; orange: no-scavengers. SB: sodium benzoate; SPB: sodium phenylbutyrate; GPB: glycerol phenylbutyrate 3%, 6% and 7% of the patients underwent liver transplantation in France, Turkey and the UK, respectively.

In terms of ammonia scavenger usage, 76% of patients were under treatment with at least one ammonia scavenger drug in Turkey, whereas 47% and 33% of them treated with one or two scavengers in France and in the UK, respectively. While, for both patients in Turkey and in the UK, dual therapy with a combination of sodium benzoate and sodium/glycerol phenylbutyrate was favoured, almost 30% of the patients were only on sodium benzoate in France (Figure 6).

## 4. Discussion

This audit, using an online survey, represents the largest collection of data for OTCD, providing an overview of 495 patients, reporting key patient characteristics including the diagnosis and management from three large countries. While there have been large international datasets involving all types of UCDs, there are no reports focusing on OTCD, which provides a similar audit and compares data from different countries [27]. 

Turkey has a larger population size compared to France and the UK, which have almost the same number of inhabitants. However, the number of patients reported from Turkey is lower than the two other countries. This may be related to the geographical distribution of the metabolic centres in Turkey. While almost two thirds of the metabolic centres are located in the three largest cities (Ankara, Istanbul and Izmir) in Turkey, only 9 of the 78 remaining cities have metabolic centres. This may cause delays and disruptions in diagnosis and could explain the lower number of patients in Turkey, whilst there are a higher number of metabolic centres compared to the UK and France. 

The relatively similar age distribution of patients in France and the UK may be related to the longer history of the identification and treatment of patients with urea cycle disorders in these countries compared with Turkey, where patients tended to be younger. Although Turkey’s population is growing older, it is still one of the youngest countries in Europe, which is consistent with the higher numbers of infantile patients compared to the other two countries. Relatively low numbers of adult patients in Turkey may also be related to the lack of adult metabolic centres and transition clinics in Turkey. At present, significant numbers of adult patients with inborn errors of metabolism (IEM) are cared for by paediatricians within paediatric services in Turkey. Awareness of IEM within the adult medical community remains limited and the dedicated work of the paediatricians could potentially still not be enough. Increased awareness of these conditions amongst all clinicians is crucial to accelerate diagnosis and appropriate management.

OTCD has a wide phenotypic spectrum in patients, ranging from the classical neonatal presentation, often leading to long-term disability and even death, to asymptomatic carrier status [28]. In our study, almost one third of patients were males in all three countries. While the percentage of symptomatic females was higher than asymptomatic females both in Turkey and France, asymptomatic females constituted approximately 40% of total patients in the UK. A longitudinal study of eight UCDs conducted by the Urea Cycle Disorders Consortium (UCDC) reported 300 OTCD patients, of whom 67 were males (22%) and 233 females (78%) [29]. A recent report based on UCDC and the European Registry and Network for Intoxication Type Metabolic Diseases (E-IMD) revealed a similar variability in the proportion of asymptomatic female patients in different geographical locations, whereas the percentages were 40.9% in North America and 25.3% in Europe [30]. These differences may be associated with varying molecular genetic heterozygote testing for at-risk female relatives in different countries. 

Several recent studies suggest that some heterozygous females may be pauci-symptomatic: while they may never have hyperammonaemia or present with altered neurological status, they may, in fact, have differences in cognitive ability, such as deficits in executive functioning, fine-motor ability, cognitive flexibility and inhibition ability, particularly after cognitive challenges [31]. Therefore, asymptomatic patients should be carefully evaluated and followed-up for underlying neurocognitive capacity differences [32].

Although most symptomatic females reported to have a late-onset presentation, around 3% of females had their first symptoms in the neonatal period. However, neonatal-onset presentation in males also showed variability, as it was higher in North America compared to Europe [30]. In a previous single-centre cohort study from France, neonatal presentation was found in 30% of total OTCD patients, while in our study, this was reported in 19% of patients in France [14]. Likewise, a single centre study from Turkey revealed that almost 10% of patients had neonatal presentation, whereas, this is reported as 12% in Turkey in our study [33]. Neonatal presentation was reported only in 7% of the UK patients in our survey. In all three countries, urea cycle disorders are not a part of neonatal screening programmes; therefore, the number of cases can be underestimated. 

As family history highlights X-linked inheritance for OTCD, it is mandatory with suspected patients to take a careful family history investigating occurrences in the family. Therefore, it is also very important to ask unexplained foetal/neonatal deaths, neurological disorders and/or protein avoidance in the family. The presence of family history was variable in three countries, whereas it was reported in 36%, 45% and 60% of the patients from France, Turkey and the UK, respectively. This variation may be associated with the differences in at-risk family screening in these three countries. 

Similar to previous reports, patients mostly presented with acute neurological symptoms accompanied by unexplained vomiting [14,34]. Although OTCD patients commonly manifest with episodes of hepatocellular injury, liver dysfunction and acute liver failure in the disease course, this was reported as an initial manifestation in only around 10% in all three countries [15,35]. Chronic symptoms including headache and protein aversion were only recorded in the UK, which has an older patient cohort and insidious symptoms such as weakness, poor feeding, abdominal pain and fatigue were stated by Turkey, which has a younger population. 

While genetic analysis has been suggested as the gold standard for diagnostic confirmation, measurement of OTC enzymatic activity in plasma, liver or intestinal mucosa has also been proposed in case of inconclusive results [16]. In accordance with this, diagnosis of OTCD was verified with genetic testing in more than 90% of patients in all three countries, with the highest enzymatic diagnosis in France. Enzymatic analysis may still play a role in cases with negative and/or unclear molecular genetic results. 

The main goals of long-term disease management are preventing hyperammonaemia episodes and maintaining metabolic control, achieving normal development and growth mainly by reducing nitrogen load through protein restriction and scavenger therapies [16]. However, the management of OTCD remains variable among centres and across countries, and our results clearly demonstrate the wide range of decisions and approaches. A survey assessing clinicians’ current perspectives and practices in urea cycle disorder management showed that there are different opinions even in the USA, and illustrated the high need for international guidelines [36]. A cross-sectional study from 41 European Inherited Metabolic Disorder (IMD) centres highlighted that the UK tended to give more total protein to UCD patients than other European countries, particularly in infancy [37]. In line with that, protein restriction was reported in less than half of the patients in the UK, whilst it was almost 1.5- and 2-fold higher in France and Turkey, respectively. It has been shown that diets are influenced by cultural preferences, geographical locations and socioeconomic factors. These factors are also important for dietary treatment adherence and compliance and can be further evaluated in a more detailed study.

Ammonia scavengers have been suggested as the mainstay of therapy by enhancing the removal of nitrogenous residues and re-establishing the nitrogen balance. Sodium benzoate conjugates with glycine to form hippurate, and sodium or glycerol phenylbutyrate, precursors of sodium phenylacetate conjugates with glutamine to form phenylacetylglutamine, which are both eliminated by the kidneys [38,39,40]. In Turkey, the total use of ammonia scavengers was reported to be higher than in France and in the UK, whereas almost one third of the patients were under dual therapy. In France, monotherapy with sodium benzoate therapy was found to be higher than the other two countries. Two thirds of patients were not on scavengers in the UK, which is consistent with the higher number of asymptomatic patients. 

Liver transplantation is recommended for consideration in severe patients without sufficient response to standard treatment and with poor quality of life, without severe neurological damage and, ideally, while in a stable metabolic condition [16,41]. However, even mortality and morbidity have been significantly improving in recent decades, and liver transplantation is still not widely considered for OTCD patients [20]. Therefore, liver transplantation rates were less than 10% in all three countries. 

As a limitation of this audit, we could not look at all important aspects in detail, as we only collected overall numbers; however, a general overview has been successfully provided. Secondly, all centres in these three countries could not be involved in this audit, even though a remarkably high number of patients have been included. We could also not audit all aspects in the suggested guideline, including the clinical, biochemical and nutritional monitoring of patients, neuroimaging, cognitive outcomes and psychosocial issues.

In conclusion, this audit highlights the diversity in the current management practices compared to the suggested guidelines in OTCD, with a special focus on a comparison of three different countries’ perspectives. These variations in clinical practices may also be related to the wide heterogeneity of phenotypes in different cohorts. This report will be helpful to provide improvements in the practice of OTCD and quality of patient care, as well as leading to closer alignment in data collection for future national and multi-national audits.

## Data Availability

The data included in this publication are available on request from the corresponding author.

## Supplementary Materials

The following supporting information can be downloaded at: https://www.mdpi.com/article/10.3390/life12111721/s1, Table S1: Questions of the online survey.
