# Supplementary material for: Three-Country Snapshot of Ornithine Transcarbamylase Deficiency
Source: Life (Basel). 2022 Oct 27;12(11):1721. doi: 10.3390/life12111721 (PMC9695856; doi:10.3390/life12111721)
Supplement: Supplementary file 1 [file life-12-01721-s001.zip › life-1948687-supplementary.pdf]

Table S1: Questions of the online survey

OTC Deficiency Patient Population Snapshot<br>Please only include alive patients into the survey
**1. Where is your medical centre?**<br><br>**UK**<br>**Turkey**<br>**France**
**2. What is your clinical centre's name?**
**3. How many OTC deficiency cases are followed up in your clinical centre?**<br>**Total number:**<br>**Male:**<br>**Female asymptomatic:**<br>**Female symptomatic:**
**4. Please give the numbers of your patients in each age group**<br>**0-6 years:**<br>**6-12 years:**<br>**12-18 years:**<br>**>18 years:**
**5. What are the three most common initial symptoms for these patients?**
**6. How many of these patients had their initial symptom in the first month of life?**
**7. How many of these patients have family history?**
**8. How many of these patients were diagnosed with enzyme activity?**
**9. How many of these patients were diagnosed with mutation analysis?**
**10. How many of these patients are on a protein restricted diet?**
**11. How many of these patients are on nitrogen scavengers?**<br>**Only Sodium benzoate:**<br>**Only Sodium or glycerol phenylbutyrate:**<br>**Both:**
**12. How many of these patients had liver transplantation?**
